# Infectious Bronchitis Virus Nsp14 Degrades JAK1 to Inhibit the JAK-STAT Signaling Pathway in HD11 Cells

**DOI:** 10.3390/v14051045

**Published:** 2022-05-14

**Authors:** Peng Ma, Kui Gu, Hao Li, Yu Zhao, Chao Li, Renqiao Wen, Changyu Zhou, Changwei Lei, Xin Yang, Hongning Wang

**Affiliations:** 1 Animal Disease Prevention and Food Safety Key Laboratory of Sichuan Province, College of Life Sciences, Sichuan University, Chengdu 610064, China; 2019322040023@stu.scu.edu.cn (P.M.); gukui0404@stu.scu.edu.cn (K.G.); 2018222040044@stu.scu.edu.cn (H.L.); 2020322040035@stu.scu.edu.cn (Y.Z.); lichaomeet@gmail.com (C.L.); 2020222040034@stu.scu.edu.cn (R.W.); 2019322040038@stu.scu.edu.cn (C.Z.); leichangwei@scu.edu.cn (C.L.); yangxin0822@scu.edu.cn (X.Y.); 2 Key Laboratory of Bio-Resource and Eco-Environment of Ministry of Education, College of Life Sciences, Sichuan University, Chengdu 610064, China

**Keywords:** infectious bronchitis virus (IBV), nonstructural protein 14 (Nsp14), IFN-γ, Janus kinase 1 (JAK1), JAK-STAT signaling pathway

## Abstract

Coronaviruses (CoVs) are RNA viruses that can infect a wide range of animals, including humans, and cause severe respiratory and gastrointestinal disease. The Gammacoronavirus avian infectious bronchitis virus (IBV) causes acute and contagious diseases in chickens, leading to severe economic losses. Nonstructural protein 14 (Nsp14) is a nonstructural protein encoded by the CoV genome. This protein has a regulatory role in viral virulence and replication. However, the function and mechanism of IBV Nsp14 in regulating the host’s innate immune response remain unclear. Here we report that IBV Nsp14 was a JAK-STAT signaling pathway antagonist in chicken macrophage (HD11) cells. In these cells, Nsp14 protein overexpression blocked IBV suppression induced by exogenous chIFN-γ treatment. Meanwhile, Nsp14 remarkably reduced interferon-gamma-activated sequence (GAS) promoter activation and chIFN-γ-induced interferon-stimulated gene expression. Nsp14 impaired the nuclear translocation of chSTAT1. Furthermore, Nsp14 interacted with Janus kinase 1 (JAK1) to degrade JAK1 via the autophagy pathway, thereby preventing the activation of the JAK-STAT signaling pathway and facilitating viral replication. These results indicated a novel mechanism by which IBV inhibits the host antiviral response and provide new insights into the selection of antiviral targets against CoV.

## 1. Introduction

Infectious bronchitis virus (IBV) is a single-stranded positive-sense RNA virus that belongs to the *Gammacoronavirus* genus, family *Coronaviridae* [1,2]. IBV causes infectious bronchitis (IB), which is an acute infectious disease that occurs in chickens worldwide [3]. The whole sequence of IBV is approximately 27.6 kb, wherein ORF1a and ORF1ab encode nonstructural proteins (Nsps) [4]. The IBV genome also encodes four structural proteins that comprise the infectious virion: spike (S), envelope (E), membrane (M), and nucleocapsid (N) proteins [5]. Nonstructural proteins are involved in host cell infection by coronaviruses (CoVs), including viral genome replication, which also antagonizes innate immunity [6].

Nonstructural protein 14 (Nsp14) is commonly acknowledged as a major functional protein in CoVs [7]. It performs an essential function in viral replication and transcription [8,9]. Nsp14 is a bifunctional enzyme that exhibits N7-methyltransferase (N7-MTase) and 3′–5′ exoribonuclease (ExoN) activities [10]. The C-terminal domain’s N7-MTase participates in RNA cap modification, and the 5′ terminal cap structure of viral mRNA assists in translation and host defense evasion [11]. The N-terminal 3′–5′ ExoN acts as a proofreader for preventing detrimental mutagenesis [12]. Therefore, the Nsp14–Nsp10 complex excises mismatched nucleotides from dsRNA [11,13]. Importantly, CoV Nsp14 is implicated in host–virus interactions that decrease host innate immunity in addition to supporting viral genome replication.

During viral infection of host cells, the antiviral response is mediated by the interferon signaling pathway [14]. Interferons, including type I (IFN-α and IFN-β), type II (IFN-γ), and type III (IFN-λ), play critical roles in the host immune response to viral infection [15]. Interferon’s antiviral function begins with binding to receptors on the cell membrane [16]. Type I IFNs bind to their corresponding receptors (IFNAR1/IFNAR2), which connect Tyk2 and JAK1. As Tyk2 and JAK1 are activated, they phosphorylate signal transducers and transcription factors STAT1 and STAT2 [15,17]. STAT1 and STAT2 form a complex with IRF9 (ISGF3) and enter the nucleus to transactivate promoters of antiviral gene expression [15]. Type II interferon (IFN-γ) binds to the interferon type II receptor (IFNGR) and then participates in the phosphorylation of JAK1 and JAK2 [18]. The phosphorylated form of STAT1, which is a homodimer called the gamma-activated factor (GAF), translocates into the nucleus and activates the transcription of interferon-stimulated genes (ISGs) by binding to the IFN-γ-activated sequence (GAS) [19,20]. Therefore, the IFN-activated JAK-STAT signaling pathway has a critical role in innate immunity against viruses. However, the interaction between the IBV nonstructural protein and the JAK-STAT signaling pathway remains poorly understood.

Herein, we explored how IBV Nsp14 antagonizes the host’s type II interferon-activated JAK-STAT signaling pathway to activate the antiviral response. We showed that the IBV Nsp14 protein inhibits the chIFN-γ signaling pathway and its downstream gene expression. Nsp14 interacts with chicken JAK1 (chJAK1), an adaptor of JAK-STAT signaling, to degrade via the autophagy pathway. Our findings demonstrate the regulation and function of Nsp14 in antagonizing host innate antiviral responses to support IBV infections.

## 2. Materials and Methods

### 2.1. Cells, Viruses, and Antibodies

Chicken HD11 and DF-1 cells were cultured in Dulbecco’s modified Eagle’s medium (DMEM, Cellmax, Beijing, China) supplemented with 10% fetal bovine serum (FBS, Cellmax) and 100  IU/mL penicillin–streptomycin solution (Cellmax). All cells were cultured in an incubator at 37 °C in 5% CO_2_.

The IBV Beaudette strain (GenBank: DQ001339) was kindly gifted by Prof. Ding-Xiang Liu, South China Agricultural University. 

Mouse monoclonal antibodies specific for Flag and β-actin, rabbit polyclonal anti-Myc antibodies, CoraLite488-conjugated donkey anti-mouse IgG (H + L), CoraLite594-conjugated donkey anti-rabbit IgG (H + L), HRP-conjugated Affinipure donkey anti-mouse IgG (H + L), and HRP-conjugated Affinipure donkey anti-rabbit IgG (H + L) were purchased from Proteintech (Wuhan, China). Mouse monoclonal anti-IBV N protein antibodies were from Novus Biologicals (Littleton, CO, USA). Rabbit polyclonal anti-chJAK1 and anti-chSTAT1 antibodies were from Abmart (Shanghai, China) and Bioss (Beijing, China), respectively.

### 2.2. Virus Infection Assay

The HD11 cells (5 × 10^5^ cells/mL) were seeded in 6-well plates and cultured for 12 h before being infected with IBV at a multiplicity of infection (MOI) of 5 and incubated in 5% CO_2_ at 37 °C for 2 h. Subsequently, the cells were washed with phosphate-buffered saline (PBS; Cellmax, China) before being cultured in DMEM supplemented with 3% FBS at 37 °C in 5% CO_2_. 

### 2.3. Transfection and Dual-Luciferase Reporter Assays

The HD11 or DF-1 cells (5 × 10^5^ cells/mL) were grown for 12 h in 6-well plates. Using Lipofectamine 8000 system (Beyotime, Beijing, China), the cells were transfected for 24 h with 200 ng reporter plasmid (pchGAS-luc) or 10 ng pRL-TK (as a control) and stimulated with 100 ng/mL chIFN-γ for 6 h. Then, using dual-luciferase assays, the whole-cell extracts were prepared for examination. Activities of the reporter genes, such as firefly luciferase and renilla luciferase, were measured using a dual-luciferase reporter 100 assay system (Beyotime, Beijing, China), as directed by the manufacturer.

### 2.4. Confocal Fluorescence Microscopy

To determine whether Nsp14 co-localizes with chJAK1, the HD11 cells were planted on glass coverslips and grown overnight in 35 mm cell culture dishes. The cells were transfected with pcDNA3.1-Flag-Nsp14 and pCMV-Myc-chJAK1 for 24 h and washed three times with cold PBS. They were treated for 15 min at room temperature with 4% paraformaldehyde. Then, the cells were incubated with anti-Flag antibodies and rabbit polyclonal anti-Myc primary antibodies (Proteintech) (1:100) for 1 h. 

To explore the effect of Nsp14 on nuclear translocation of chSTAT1, HD11 cells were transfected with pcDNA3.1-Flag-Nsp14 for 24 h, stimulated with chIFN-γ (100 ng/mL) for 30 min before being washed three times with cold PBS, and treated for 15 min at room temperature with 4% paraformaldehyde. The cells were then incubated with anti-Flag (Proteintech) and anti-chSTAT1 (Bioss) antibodies (1:100) for 1 h. 

All the cells were washed with PBS and incubated with secondary antibodies (CoraLite488-conjugated donkey anti-mouse IgG (H + L) and CoraLite594-conjugated donkey anti-rabbit IgG (H + L) (1:200)) for 1 h. Then, the cells were dyed with DAPI (Beyotime) for 10 min and washed with cold PBS. The images were captured using a laser scanning confocal microscope (Zeiss, Jena, Germany).

### 2.5. Co-Immunoprecipitation Assays

The HD11 cells (5 × 10^5^ cells/mL) cultured in 6-well plates were co-transfected with the following plasmids: pcDNA3.1-Flag-Nsp14, pCMV-Myc-chJAK1, pCMV-Myc-chJAK2, pCMV-Myc-chSTAT1, pcDNA3.1-Flag-Nsp14-FL (full length 522 aa), pcDNA3.1-Flag-Nsp14-N (1–288 aa), or pcDNA3.1-Flag-Nsp14-C (289–522 aa) using Lipofectamine 8000. The transfected cells were lysed on ice for 30 min after being transfected for 24 h with RIPA lysis solution (500 μL/well) containing 1 mM PMSF (Beyotime). The lysates were centrifuged for 30 min at 12,000 g. Approximately 25% of the supernatant was subjected to input assays, and the remaining supernatant was utilized for the co-immunoprecipitation (Co-IP) test with an anti-Flag agarose affinity gel (Beyotime). The agarose affinity gel (50 µL) was then centrifuged for 30 s at 4 °C to remove the solution before being rinsed with cold TBS. On a revolving platform, the agarose affinity gel was added to the cell lysate while being gently rocked at 4 °C overnight. The protein samples were analyzed using Western blotting after the agarose affinity gel was washed with cold TBS.

### 2.6. Western Blot

For 30 min on ice, the cultured cells were lysed using a RIPA lysis buffer. After adding the SDS-PAGE sample loading buffer, the samples were heated for 5 min at 95 °C. The program was run on a 10% SDS-PAGE at 120 V for 100 min. The protein was transferred to a PVDF membrane (pore size, 0.22 μM; Invitrogen, Carlsbad, CA, USA) for 1.5 h at 200 mA. Then, the membrane was blocked for 1 h at room temperature with a blocking solution containing 1% BSA. It was then incubated overnight at 4 °C with a primary antibody (1:1000). The membrane was then washed three times and subsequently incubated with a secondary antibody (1:2000) for 1 h at room temperature. After three washes, protein bands were detected using the BeyoECL Moon chemiluminescent system (Beyotime).

### 2.7. Quantitative Real-Time Polymerase Chain Reaction 

Total RNA from the samples was isolated using the RNA-easy isolation reagent (Vazyme Biotech Co., Ltd., R701, Nangjing, China). The RNA (1 μg) was further processed using the RevertAid First Strand cDNA Synthesis kit (Thermo Fisher, Waltham, MA, USA, K1621) to produce cDNA according to the manufacturer’s instructions.

A Bio-Rad system (Hercules, CA, USA) was used to perform quantitative real-time polymerase chain reaction (qRT-PCR). To perform qRT-PCR, 1 µL of cDNA as mixed with 10 µL of ChamQ Universal SYBR qPCR Master Mix (Vazyme Biotech Co., Ltd.), 0.5 µL of forward/reverse primers, and 8 µL of ddH_2_O in a total 20 µL volume. The samples were heated to 95 °C for 10 min before undergoing 40 cycles of PCR, which included 10 s at 95 °C, 20 s at 60 °C, and 15 s at 72 °C. The IBV genome copy number was quantified using our previously established method. The formula of the standard curve was y = 39.760 − 3.909x, R^2^ = 0.997. To normalize the gene expression using the 2^−ΔΔCT^ formula, the glyceraldehyde-3-phosphate dehydrogenase gene (GAPDH) was employed as a housekeeping gene. Table 1 lists the primers used in this study.

### 2.8. Statistical Analysis

Statistical analysis was performed using GraphPad Prism 5 software (Mann–Whitney test; GraphPad Software Inc., La Jolla, CA, USA). Statistical significance was set at *: *p* < 0.05, **: *p* < 0.01, ***: *p* < 0.001, ns: not significant.

## 3. Results

### 3.1. Nsp14 Protein Antagonized Type II Interferon Anti-IBV

**Figure 1:**
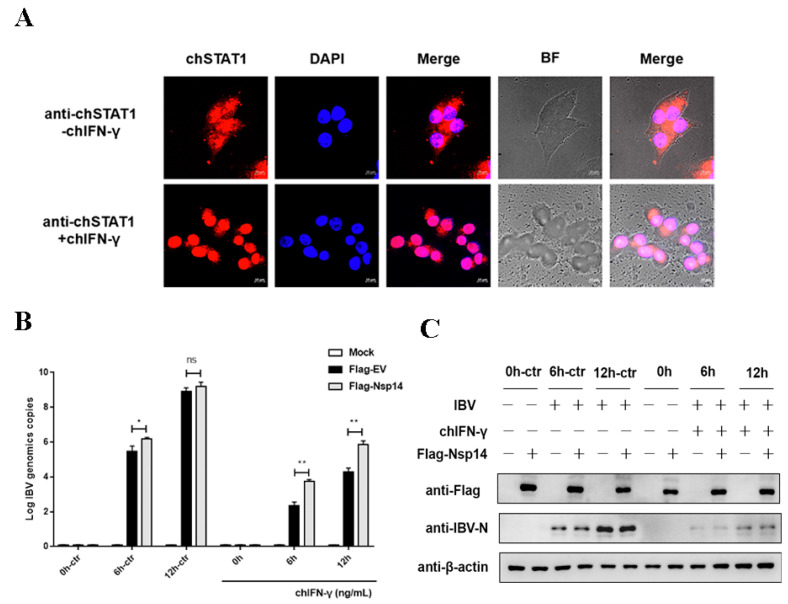
IBV Nsp14 protein antagonized type II interferon anti-IBV. (**A**) HD11 cells were stimulated with 100 ng/mL chIFN-γ at 30 min. chSTAT1 protein was detected using an indirect immunofluorescence assay. The cell nucleus was counterstained with DAPI. chSTAT1 nuclear accumulation was determined using confocal fluorescence microscopy. (**B**) HD11 cells were transfected with pcDNA3.1-Flag-Nsp14 or pcDNA3.1-Flag (as an empty vector) at 24 h after IBV infection (MOI = 5) and stimulated with chIFN-γ (1000 ng/mL) for 12 h. qRT-PCR was used to detect IBV N mRNA expression. (**C**) Western blotting was used to detect the IBV N protein level. Data were presented as means ± SD. * = *p* < 0.05, ** = *p* < 0.01, ns = not significant.

To determine whether IBV antagonized the host’s interferon-activated JAK-STAT signaling pathway which activates the antiviral response in the chicken macrophages (HD11 cells). To verify the effect of the chicken interferon-gamma (chIFN-γ)-activated JAK-STAT signaling pathway on IBV replication, the HD11 cells were treated with chIFN-γ. Nuclear translocation of chicken STAT1 (chSTAT1) in chIFN-γ-treated HD11 cells was used to investigate the biological activity and responses of chIFN-γ. The results showed that chIFN-γ stimulated the nuclear translocation of chSTAT1 in the HD11 cells and, thus, accumulated in the nucleus (Figure 1A). The HD11 cells were transfected with IBV Nsp14 for 24 h, stimulated with chIFN-γ at 1000 ng/mL, and infected with IBV Beaudette (MOI = 5). The viral replication levels were determined at 0, 6, and 12 h post-infection (hpi). IBV replication was dramatically reduced after the chIFN-γ treatment. After the Nsp14 transfection, chIFN-γ inhibition was weakened and IBV replication was increased at the mRNA level compared with the control group (Figure 1B). After the chIFN-γ treatment, the level of IBV nucleocapsid protein was also examined. IBV Nsp14 decreased the chIFN-γ anti-IBV replication at the protein level according to the findings (Figure 1C). These data suggested that the IBV Nsp14 protein significantly inhibited the antiviral effect of chIFN-γ.

### 3.2. Nsp14 Protein Inhibited ChIFN-γ-Activated Signaling and Downstream Gene Expression

**Figure 2:**
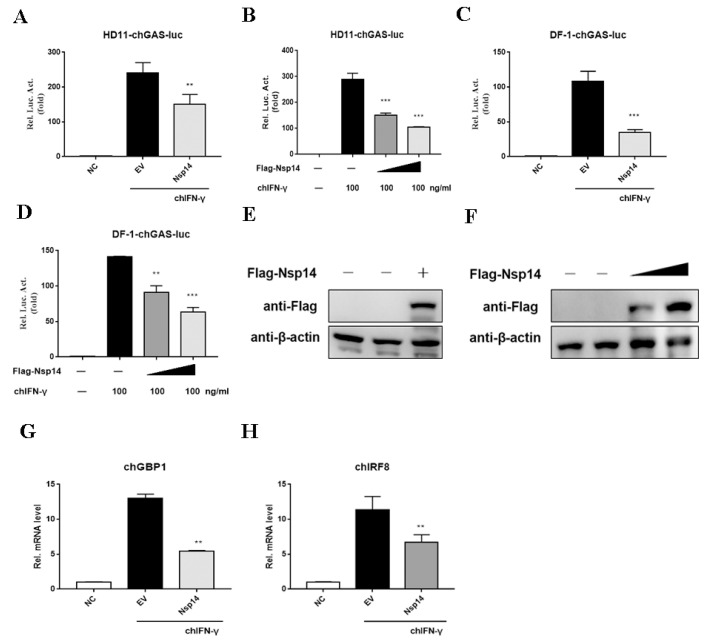
IBV Nsp14 inhibited chIFN-γ signaling and downstream gene expression. (**A**,**B**) HD11 or DF-1 cells were co-transfected with pcDNA3.1-Flag-Nsp14 (1 μg), pchGAS-luc (200 ng), and pRL-TK (10 ng). After 24 h, the cells were stimulated with chIFN-γ (100 ng/mL) for 6 h. Then, luminescence was detected using dual-luciferase reporter assays. (**C**,**D**) HD11 or DF-1 cells were co-transfected with increasing concentrations pcDNA3.1-Flag-Nsp14 (1 μg) and pcDNA3.1-Flag-Nsp14 (2 μg). After 24 h, the cells were stimulated with chIFN-γ (100 ng/mL) for 6 h. Luminescence was detected using dual-luciferase reporter assays. (**E**,**F**) Western blotting was used to detect the IBV Nsp14 protein level. (**G**,**H**) HD11 cells were transfected with pcDNA3.1-Flag-Nsp14 (1 μg). After 24 h, the cells were stimulated with chIFN-γ (100 ng/mL) for 6 h. chGBP1 and chIRF8 mRNA levels were detected using the qRT-PCR assay. Data were presented as means ± SD. ** = *p* < 0.01, *** = *p* < 0.001.

Next, to test whether the Nsp14 protein repressed IFN-γ-activated JAK-STAT signaling, we constructed the reporter gene containing the chicken GAS (chGAS). The HD11 cells were co-transfected with pchGAS-luc and an increasing concentration of the Flag-Nsp14 plasmid. Then, the cells were stimulated with chIFN-γ for 6 h before being subjected to the dual luciferase assay. This assay revealed that the Nsp14 protein inhibited the chIFN-γ-induced chGAS promoter activity (Figure 2A). The dose-dependent assay revealed that the Nsp14 protein dose-dependently inhibited chGAS promoter activation (Figure 2B). We also verified consistent results in the chicken fibroblast cell line DF-1 in which Nsp14 inhibited chIFN-γ-activated JAK-STAT signaling in a dose-dependent manner (Figure 2C,D). The expression of Nsp14 protein was detected (Figure 2E,F). Next, whether the Nsp14 protein interfered with chIFN-γ-induced downstream gene transcription was verified. The HD11 cells were transfected with Nsp14 and then stimulated with chIFN-γ. *chGBP1* (Figure 2G) and *chIRF8* expression levels (Figure 2H) were inhibited by the Nsp14 protein. Hence, the Nsp14 protein inhibited chIFN-γ-activated signaling and downstream gene mRNA synthesis. The data demonstrated that Nsp14 specifically inhibited the chIFN-γ-activated JAK-STAT signaling pathway in a dose-dependent manner.

### 3.3. Nsp14 Protein Inhibited ChSTAT1 Nuclear Translocation

**Figure 3:**
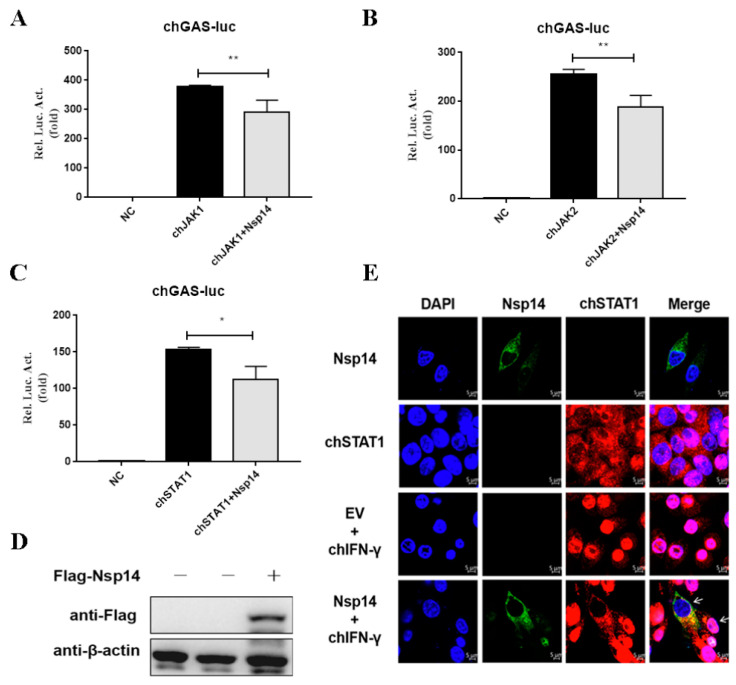
IBV Nsp14 inhibited chIFN-γ-mediated chSTAT1 nuclear translocation. (**A**–**C**) HD11 cells were co-transfected with pcDNA3.1-Flag-Nsp14 (1 μg) or pCMV-Myc-chJAK1 (500 ng), pCMV-Myc-chJAK2 (500 ng), and pCMV-Myc-chSTAT1 (500 ng). After 24 h, luminescence was detected using dual-luciferase reporter assays. (**D**) Western blotting was used to detect the IBV Nsp14 protein level. (**E**) HD11 cells were transfected with pcDNA3.1-Flag-Nsp14 (1 μg) for 24 h. The cells were stimulated with chIFN-γ (100 ng/mL) for 30 min. chSTAT1 protein was detected using an indirect immunofluorescence assay. chSTAT1 nuclear accumulation was determined using confocal fluorescence microscopy. Data were presented as means ± SD. * = *p* < 0.05, ** = *p* < 0.01.

To confirm whether Nsp14 protein inhibited adapter proteins of the chIFN-γ activated JAK-STAT signaling pathway, Nsp14 or an empty vector, as well as adaptor proteins chJAK1, chJAK2, and chSTAT1, were transfected into the HD11 cells, which were subsequently stimulated with chIFN-γ for 6 h. Activation of JAK-STAT signaling by chJAK1 (Figure 3A), chJAK2 (Figure 3B), and chSTAT1 (Figure 3C) was greatly inhibited by the Nsp14 protein. The expression of Nsp14 protein was detected (Figure 3D). To explore whether the Nsp14 protein regulated chSTAT1 translocation from the cytoplasm to the nucleus, the HD11 cells were transfected with Nsp14 or an empty vector and treated with chIFN-γ for 30 min. Then, chSTAT1 localization was analyzed. Immunofluorescence analyses showed that HD11 cells expressing the empty vector exhibited substantial chSTAT1 nuclear accumulation, whereas those expressing IBV Nsp14 displayed rare chSTAT1 nuclear accumulation (Figure 3E). These data indicated that IBV Nsp14 inhibited chIFN-γ-triggered chSTAT1 nuclear translocation.

### 3.4. Nsp14 Protein Interacts with chJAK1 Protein

**Figure 4:**
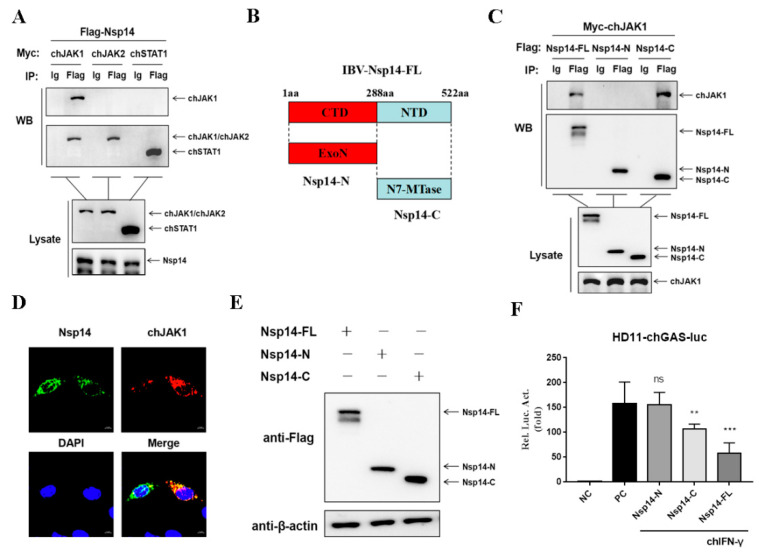
IBV Nsp14 interacted with chJAK1. (**A**) HD11 cells were co-transfected with pCMV-Myc-chJAK1 (1 μg), pCMV-Myc-chJAK2 (1 μg), and pCMV-Myc-chSTAT1 (1 μg) and either pcDNA3.1-Flag-Nsp14 (1 μg) or pcDNA3.1-Flag-EV (1 μg). The transfected cells were harvested. Co-IP was performed using an anti-Flag antibody (1:100). The precipitated proteins were analyzed using Western blotting by using anti-Myc antibodies. (**B**) Diagram of the N-terminal and C-terminal of Nsp14. (**C**) HD11 cells were co-transfected with pcDNA3.1-Flag-Nsp14-FL (1 μg), pcDNA3.1-Flag-Nsp14-N (1 μg), and pcDNA3.1-Flag-Nsp14-C (1 μg) and either pCMV-Myc-chJAK1 (1 μg) or pCMV-Myc-EV (1 μg). The transfected cells were harvested. Co-IP was performed using an anti-Flag antibody (1:100). The precipitated proteins were analyzed using Western blotting by using anti-Myc antibodies. (**D**) HD11 cells were transfected with pcDNA3.1-Flag-Nsp14 (1 μg) and pCMV-Myc-chJAK1 (1 μg) for 24 h. The cells were fixed and subjected to an indirect immunofluorescence assay to detect colocalization. The colocalization of Nsp14 and chJAK1 was visualized using confocal fluorescence microscopy. (**E**,**F**) HD11 cells were transfected with pcDNA3.1-Flag-Nsp14-FL (1 μg), pcDNA3.1-Flag-Nsp14-N (1 μg), and pcDNA3.1-Flag-Nsp14-C (1 μg). After 24 h, the cells were stimulated with chIFN-γ (100 ng/mL) for 6 h. The protein was detected using Western blotting, and luminescence was detected using dual-luciferase reporter assays. Data were presented as means ± SD. ** = *p* < 0.01, *** = *p* < 0.001, ns = not significant.

The relationship between the Nsp14 protein and adapter proteins of the chIFN-γ-activated JAK-STAT signaling pathway was then investigated. Then, the HD11 cells were co-transfected with Nsp14, chJAK1, chJAK2, or chSTAT1. Co-IP assays were used to detect the interaction. In the Co-IP tests, the Nsp14 protein interacted with the chJAK1 protein but not with the chJAK2 or chSTAT1 protein (Figure 4A). To further confirm the region of Nsp14 protein required for binding to the chJAK1 protein, two plasmids expressing the Flag-tagged truncated mutants of Nsp14 were constructed (Figure 4B). The chJAK1–Nsp14 interaction was determined using the Co-IP assays. The findings suggested that the chJAK1 protein interacted with the full length and the C-terminal region (Figure 4C). Furthermore, in the absence of chIFN-γ stimulation, confocal microscopy revealed that the Nsp14 protein co-localized with the chJAK1 protein in the cytoplasm of the HD11 cells (Figure 4D). 

We further evaluated the effect of the region of Nsp14 protein on inhibiting the chIFN-γ-activated JAK-STAT signaling pathway. We co-transfected the HD11 cells with pchGAS-luc and the plasmids encoding Nsp14 (N- and C-terminal regions) or an empty vector and then stimulated the cells for 6 h with chIFN-γ. Protein expression was detected using Western blotting (Figure 4E). The finding revealed that the N-terminal of Nsp14 protein had no effect on the chGAS promoter, whereas the chGAS promoter was significantly reduced in Nsp14 full-length and C-terminal plasmid-transfected cells (Figure 4F). These findings indicated that the IBV Nsp14’s full length and C-terminal region interacted with the chJAK1 protein to block the chIFN-γ-activated JAK-STAT signaling pathway.

### 3.5. Nsp14 Protein Reduced the ChJAK1 Protein Level

**Figure 5:**
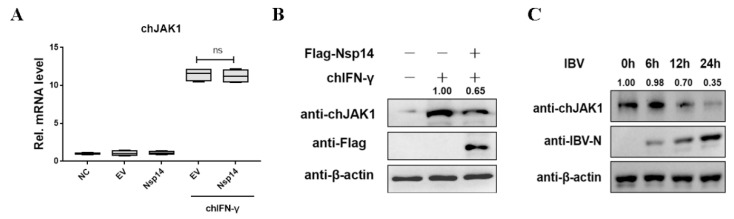
IBV Nsp14 reduces chJAK1 protein. (**A**,**B**) HD11 cells were transfected with pcDNA3.1-Flag-Nsp14 (1 μg) or pcDNA3.1-Flag-EV (1 μg). After 24 h, the cells were stimulated with chIFN-γ (100 ng/mL) for 6 h. The chJAK1 genes (**A**) were evaluated using the qRT-PCR assay, and the protein level (**B**) was detected using Western blotting. (**C**) HD11 cells were infected with IBV (MOI = 5) for 0 h, 6 h, 12 h, and 24 h. Western blotting was used to detect the chJAK1 and IBV N protein levels. Data were presented as means ± SD. ns = not significant.

Furthermore, to determine the effects of the Nsp14 protein on the JAK1 expression levels, an adapter proteins of the chIFN-γ-stimulated JAK-STAT signaling pathway, Nsp14 or an empty vector was transfected into the HD11 cells, which were then stimulated with chIFN-γ for 6 h. The effects of Nsp14 were evaluated using qRT-PCR and Western blot analysis. It was found that the overexpression of Nsp14 protein had no effect on the chJAK1 at the mRNA level (Figure 5A). Interestingly, with chIFN-γ stimulation, chJAK1 protein expression was reduced in the Nsp14-overexpressing HD11 cells (Figure 5B). To investigate the effect of IBV infection on chJAK1 expression, HD11 cells were infected with IBV for the indicated time courses, and endogenous chJAK1 was detected using an antibody. The results showed that IBV infection decreased the expression of chJAK1 proteins in HD11 cells. Taken together, the data suggested that IBV Nsp14 tended to inhibit chJAK1 protein levels.

### 3.6. Nsp14 Protein Degraded the ChJAK1 Protein via the Autophagy Pathway

**Figure 6:**
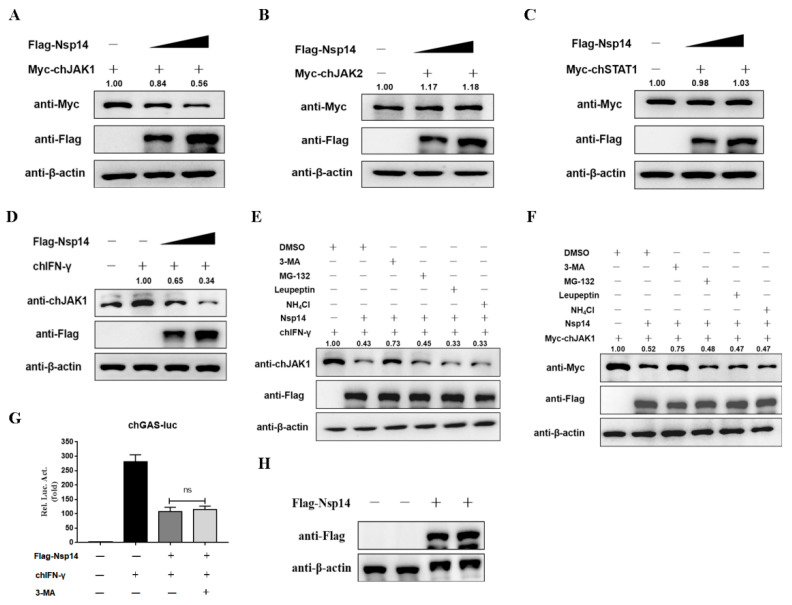
IBV Nsp14 degraded chJAK1 via the autophagy pathway. (**A**–**C**) HD11 cells were co-transfected with pcDNA3.1-Flag-Nsp14 (1 μg), pcDNA3.1-Flag-Nsp14 (2 μg) or pCMV-Myc-chJAK1 (1 μg), pCMV-Myc-chJAK2 (1 μg), and pCMV-Myc-chSTAT1 (1 μg). After 24 h, protein expression was measured using Western blotting. (**D**) HD11 cells were transfected with pcDNA3.1-Flag-Nsp14 (1 μg), pcDNA3.1-Flag-Nsp14 (2 μg), or pcDNA3.1-Flag-EV (1 μg). After 24 h, the cells were stimulated with chIFN-γ (100 ng/mL) for 6 h. Protein expression was measured using Western blotting. (**E**) HD11 cells were transfected with pcDNA3.1-Flag-Nsp14 (1 μg) or pcDNA3.1-Flag-EV (1 μg). After 24 h, the cells were stimulated with chIFN-γ (100 ng/mL) and then treated with dimethyl sulfoxide (DMSO), 3-MA (0.5 mg/mL), MG-132 (20 μM), leupeptin (400 μg/mL), and NH_4_Cl (20 mM) for 6 h. Protein expression was measured using Western blotting. (**F**) HD11 cells were co-transfected with pcDNA3.1-Flag-Nsp14 (1 μg) and pCMV-chJAK1 (1 μg). After 24 h, the cells were incubated with DMSO, 3-MA (0.5 mg/mL), MG-132 (20 μM), leupeptin (400 μg/mL), and NH4Cl (20 mM) for 6 h. Protein expression was detected using Western blotting. (**G**,**H**) HD11 or DF-1 cells were co-transfected with pcDNA3.1-Flag-Nsp14 (2 μg), pchGAS-luc (200 ng), and pRL-TK (10 ng). Meanwhile, the cells were treated with 3-MA. After 24 h, the cells were stimulated with chIFN-γ (100 ng/mL) for 6 h. Then, luminescence was detected using dual-luciferase reporter assays. Protein expression was detected using Western blotting. Data were presented as means ± SD. ns = not significant.

Notably, IBV Nsp14 reduced the chJAK1 protein level and interacted with chJAK1, but did not inhibit mRNA expression (Figure 5). Subsequently, we examined the effect of Nsp14 on chJAK1 protein. The HD11 cells were co-transfected with Nsp14 and chJAK1, chJAK2, or chSTAT1, and the protein level was measured using Western blotting. The Nsp14 protein, as expected, decreased the chJAK1 protein expression level (Figure 6A) in a dose-dependent manner, but had no effect on chJAK2 (Figure 6B) or chSTAT1 levels (Figure 6C). Moreover, we examined the potential effects of Nsp14 on the level of endogenous chJAK1 protein. The HD11 cells were transfected with Nsp14 and stimulated with chIFN-γ for 6 h. We found that levels of the endogenous chJAK1 protein were reduced by the Nsp14 protein in a dose-dependent manner (Figure 6D). 

The findings demonstrated that Nsp14 reduced the levels of both endogenous and exogenous chJAK1 protein, but did not affect its gene level, probably via degradation. Then, we explored how Nsp14 degraded chJAK1. The HD11 cells were transfected with Nsp14 and treated with an autophagy inhibitor (3-MA), a proteasome inhibitor (MG-132), or a lysosomal inhibitor (leupeptin and NH_4_Cl). The autophagy inhibitor (3-MA) blocked the degradation of both endogenous (Figure 6E) and exogenous chJAK1 proteins (Figure 6F). However, the inhibition of Nsp14 on the chGAS promoter was not diminished when cells were treated with 3-MA (Figure 6G). Protein expression was detected using Western blotting (Figure 6H). It was related to the inhibition of chSTAT1 nuclear accumulation by Nsp14. Taken together, these results suggested that IBV Nsp14 degraded both endogenous and exogenous chJAK1 proteins via the autophagy pathway.

## 4. Discussion

**Figure 7:**
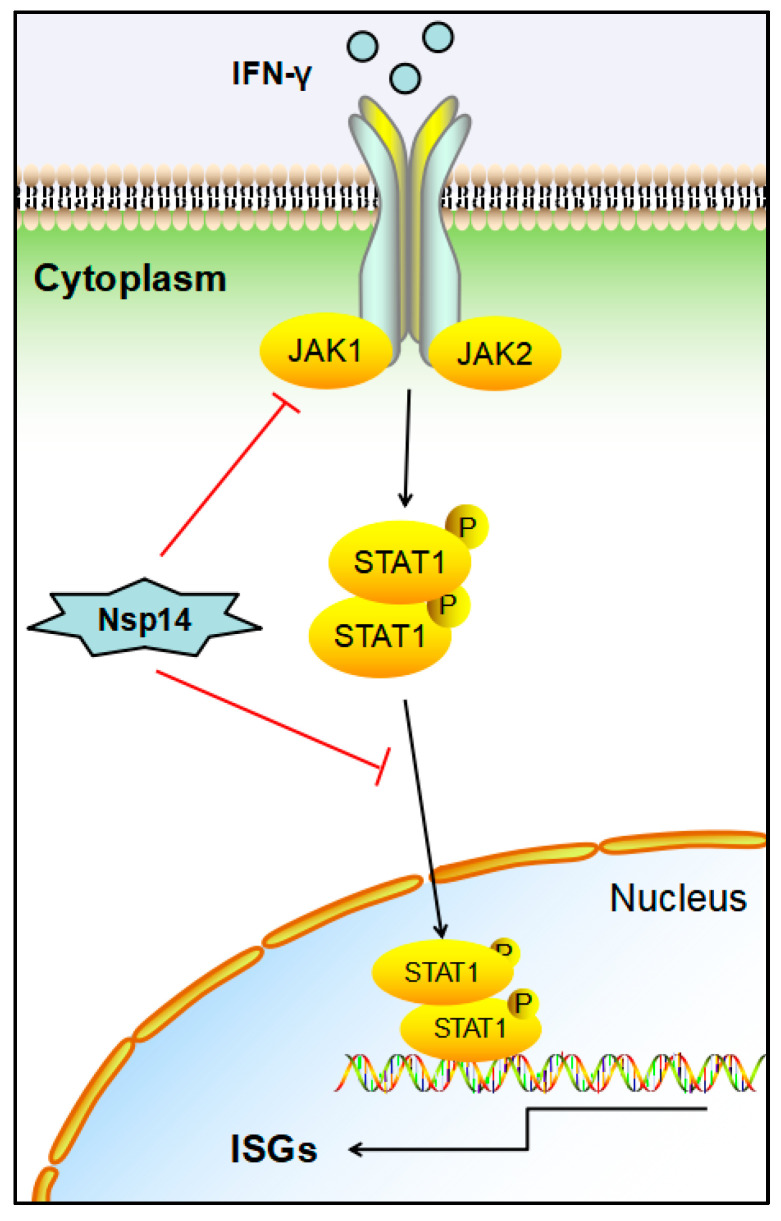
IBV Nsp14 inhibited the JAK-STAT signaling pathway. IBV Nsp14 antagonized the chIFN-γ-mediated antiviral response, which targeted chJAK1 degradation and blocked chSTAT1 nuclear translocation to inhibit the JAK-STAT signaling pathway.

Viruses have evolved various strategies that dramatically alter the host’s innate immune defenses to infect, propagate, and spread [21]. Our data revealed that IBV regulated the host’s innate immune response by inhibiting the JAK-STAT signaling pathway in chicken macrophages (HD11 cells), which facilitated viral replication. The IBV Nsp14 suppressed JAK-STAT signaling and downstream gene expression via antagonizing the chIFN-γ-mediated antiviral response. Furthermore, we found that the Nsp14 protein inhibited chJAK1-, chJAK2-, and chSTAT1-mediated activation of the JAK-STAT signaling pathway by inhibiting chSTAT1 nuclear translocation. Notably, the Nsp14 protein interacted with the chJAK1 protein, thereby promoting chJAK1 degradation via the autophagy pathway (Figure 7). In summary, this study demonstrated a mechanism by which IBV regulated innate immune responses via JAK-STAT signaling in HD11 cells.

Interferons play a significant role in host defense against CoVs [22]. Upon virus infection, pathogen-associated molecular patterns (PAMP) are recognized by host pattern recognition receptors (PRR), which activate TLR and RLR signaling pathways and produce interferon [23]. Importantly, in an early stage of viral infection, interferon is a crucial cytokine controlling viral infection and replication [16,24,25]. CoVs employ various ways to evade host interferon signaling and more efficiently replicate and produce progeny viruses in cells. During CoVs infection, the host interferon production is delayed to varying degrees [26,27,28,29,30]. Nevertheless, some CoVs are relatively resistant to IFN treatment in vitro [31,32,33]. By contrast, MERS-CoV and SARS-CoV-2 are more sensitive to IFN treatment, requiring only low IFN concentrations [29,34]. In vitro, IBV reproduction can only be prevented at higher IFN-α and IFN-β concentrations, implying that IBV is relatively resistant to type I IFN and may actively counteract type I IFN responses [35]. We found that IBV is also resistant to type II IFN treatment; the main antagonist is Nsp14 encoded by IBV. Therefore, Nsp14 may be associated with IBV antagonizing the JAK-STAT signaling pathway activated by IFN-γ.

The CoV Nsp14 was found to be involved in antagonizing host IFNs-activate the JAK-STAT signaling pathway [36]. The SARS-CoV-2 Nsp14 protein inhibits Sendai-virus-activated IFN-β and IRES promoters [29]. Stimulation with IFN-α2, IFN-β, IFN-γ, or IFN-λ1 revealed that activation of ISRE and the GAS promoter is strongly repressed by SARS-CoV-2 Nsp14 [37]. Based on this, we explored whether IBV Nsp14 inhibited chIFN-γ signaling in HD11 cells. We also found that IBV Nsp14 inhibited the chIFN-γ-activated JAK-STAT signaling pathway in a dose-dependent manner. The CoV Nsp14 protein acts as a bifunctional protein, with the ExoN domain being crucial for CoV replication proofreading activity and the N7-MTase domain being involved in viral mRNA capping [38,39]. Recombinant MHV using reverse genetic techniques demonstrated that guanine N7-methylation of CoV Nsp14 helps the virus evade the type-I-IFN-mediated immune response and pathogenesis in mice [40]. As for IBV, further exploring the role of different domains of bifocal Nsp14 in antagonizing innate immunity is necessary. Our results revealed that the N7-MTase domain at the C-terminal of Nsp14 directly inhibited chGAS promoter activity with stimulation of chIFN-γ rather than the N-terminal ExoN domain. The MHV Nsp14 N7-MTase, which isthe guanine N7-methylation of the viral RNA cap structure, is necessary to prevent viral RNA from being recognized by the cell’s innate sensors [40]. Compared with the N7-MTase domains, ExoN-deletion MHV replication does not lead to IFN expression but enhances sensitivity to IFN-β treatment [41]. In addition, ExoN and N7-MTase domains in the human CoV Nsp14 protein inhibit IFN-dependent ISG induction by inhibiting cellular translation. Even with approximately 70% amino acid similarity, IBV Nsp14 merely slightly affects translation [42]. 

Next, we investigated how IBV Nsp14 antagonized the chIFN-γ-activated JAK-STAT signaling pathway. In the pathway, when IFNs bind to specific receptors, the molecular conformation of JAKs changes, triggering their autophosphorylation or transphosphorylation and subsequent docking and phosphorylation of STATs [43,44]. The nuclear localization of STAT1 and STAT2 is mediated by tyrosine phosphorylation, which is triggered by IFN-γ-mediated homologous dimerization and IFN-α/IFN-β- and IFN-λ-mediated heterodimerization [45]. The JAK-STAT signaling pathway can be antagonized in two main ways. One is to block binding to ISRE and GAS by inhibiting STAT1 nuclear translocation. For the CoVs, SARS-CoV-2 and SARS-CoV ORF6 inhibit ISRE promoter activation by blocking STAT1 nuclear translocation but not phosphorylation [29,46,47]. However, in IBV-infected Vero cells, IBV-mediated inhibition of STAT1 nuclear translocation after IFN-β treatment is independent of accessory proteins [35]. Moreover, in our results, chGAS promoter activity was significantly inhibited by IBV Nsp14 after the overexpression of chJAK1, chJAK2, and chSTAT1 under chIFN-γ stimulation. Confocal microscopy revealed that Nsp14 overexpression blocked chSTAT1 nuclear translocation. The other way of antagonizing the JAK-STAT signaling pathway is by interacting with the adaptor protein to block its activity, for example, by inhibiting protein phosphorylation or degrading protein. SARS-CoV-2 Nsp14 induces lysosomal degradation of IFNAR1, an essential receptor for type I IFN, thereby preventing the activation of STAT transcription factors [37]. SARS-CoV-2 Nsp13 interacts with STAT1 to prevent JAK1-induced STAT1 phosphorylation [48,49]. Our assays revealed that IBV Nsp14 interacted with chJAK1 to reduce chJAK1 protein levels, but not the gene levels. Crucially, when IBV infected HD11 cells, chJAK1 protein expression was also inhibited. To further explore the mechanism of Nsp14-mediated chJAK1 degradation, we used chemical inhibitors to inhibit several methods of protein degradation. We found the role of 3-MA in restoring chJAK1 levels, demonstrating that Nsp14 degraded the chJAK1 protein via the autophagy pathway. However, further investigation into the specific autophagy mechanism involved in IBV Nsp14 degrading chJAK1 is required. Finally, IBV Nsp14 suppression of the IFN-γ-activated JAK-STAT signaling pathway was not restored by 3-MA treatment of the cells. The reason for this was that Nsp14 prevented STAT1 nuclear translocation.

## 5. Conclusions

In summary, our results revealed the molecular mechanism by which IBV Nsp14 antagonized innate immunity. Moreover, this work identified IBV Nsp14 as a chJAK1 antagonist. IBV Nsp14 targeted chJAK1 degradation and blocked chSTAT1 nuclear translocation to inhibit the JAK-STAT signaling pathway. We concluded that Nsp14 could be a potential antiviral target against the replication of all CoVs.

## Figures and Tables

**Table 1:** Primers used in this study.

Primers	Forward (5′–3′)	Reverse (5′–3′)
IBV-N	GAAGAAAACCAGTCCCAGA	TTACCAGCAACCCACAC
GAPDH	CATCACAGCCACACAGAAG	GGTCAGGTCAACAACAGAGA
chJAK1	AGATGATGAGAATGAAGGATA	ACGATGTGCTTATGAGAA
chGBP1	AAGTCCTTCCTGATGAACC	CTTGGTCTCCGCATACAC
chIRF8	CAGGACTACAACCAGGAG	ACCTTCTTTGAATTTGCCTTT

## Data Availability

Not applicable.
